# A new possible megalosauroid theropod from the Middle Jurassic Xintiangou Formation of Chongqing, People’s Republic of China and its implication for early tetanuran evolution

**DOI:** 10.1038/s41598-019-56959-x

**Published:** 2020-01-10

**Authors:** Hui Dai, Roger Benson, Xufeng Hu, Qingyu Ma, Chao Tan, Ning Li, Ming Xiao, Haiqian Hu, Yuxuan Zhou, Zhaoying Wei, Feng Zhang, Shan Jiang, Deliang Li, Guangzhao Peng, Yilun Yu, Xing Xu

**Affiliations:** 1 Chongqing Laboratory of Geoheritage Protection and Research, No. 208 Hydrogeological and Engineering Geological Team, Chongqing Bureau of Geological and Mineral Resource Exploration and Development, Chongqing, 400700 China; 2 Department of Earth Sciences, University of Oxford, Oxford, OX1 3AN UK; 3 Zigong Dinosaur Museum, Zigong, 643013 Sichuan China; 4 Chongqing Institute of Geological Survey, Chongqing, 401122 China; 5 Key Laboratory of Vertebrate Evolution and Human Origins of Chinese Academy of Sciences, Institute of Vertebrate Paleontology and Paleoanthropology, Chinese Academy of Sciences, Beijing, 100044 China; 6 CAS Center for Excellence in Life and Paleoenvironment, Beijing, China

**Keywords:** Sedimentology, Palaeontology

## Abstract

Tetanurae is a special group of theropod dinosaurs that originated by the late Early Jurassic. It includes several early-diverging groups of generally large-bodied predators (megalosauroids, allosauroids, tyrannosauroid coelurosaurs) as well as morphologically disparate small-bodied coelurosaurs, including birds. Aspects of the evolutionary history of tetanurans remain contested, including the topology of their deep phylogenetic divergences (among Megalosauroidea, Allosauroidea and Coelurosauria). We report a new theropod, *Yunyangosaurus puanensis* gen. et sp. nov., based on a fragmentary specimen recovered from the Middle Jurassic Xintiangou Formation of Chongqing, southwestern China. It shares several features uniquely with some megalosauroids (the clade of megalosaurids + spinosaurids + piatnitzkysaurids), such as prominent rims around the anterior articular surfaces of cervical centra and bifurcated anterior dorsal neural spines (present in piatnitzkysaurids). Nevertheless, it also shows several features that are rare or absent among megalosauroids and more crownward tetanurans, including prominent spinopostyzgopophyseal laminae (also present in non-tetanurans and metriacanthosaurid allosauroids), flat anterior articular surfaces of the cervical centra (also present in piatnitzkysaurids and some earlier-diverging tetanurans), and the presence of a posterior pneumatic foramen or fossa (absent in most tetanurans, but sporadically present in some cervical vertebrae of piatnitzkysaurids). *Yunyangosaurus* therefore presents a combination of derived and apparently primitive character states that are not seen in other theropods. This suggests that patterns of morphological evolution associated with deep tetanuran divergences were more complex than currently recognized, with implications for understanding the character evolution in theropods.

## Introduction

**Figure 1:**
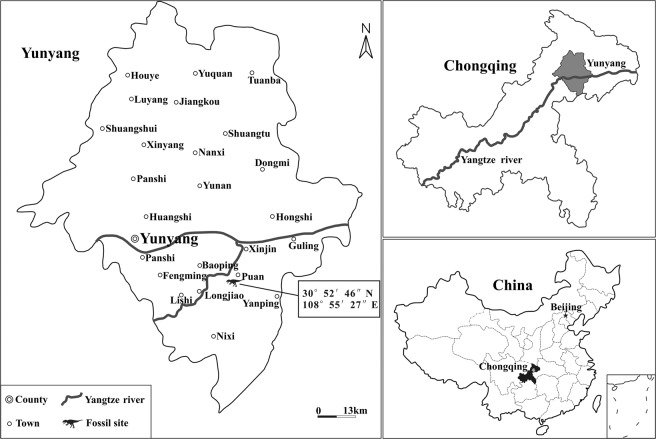
Geographical map indicating the location of the fossil site within the Yunyang County, Chongqing Municipality, China. Modified from Li.

**Figure 2:**
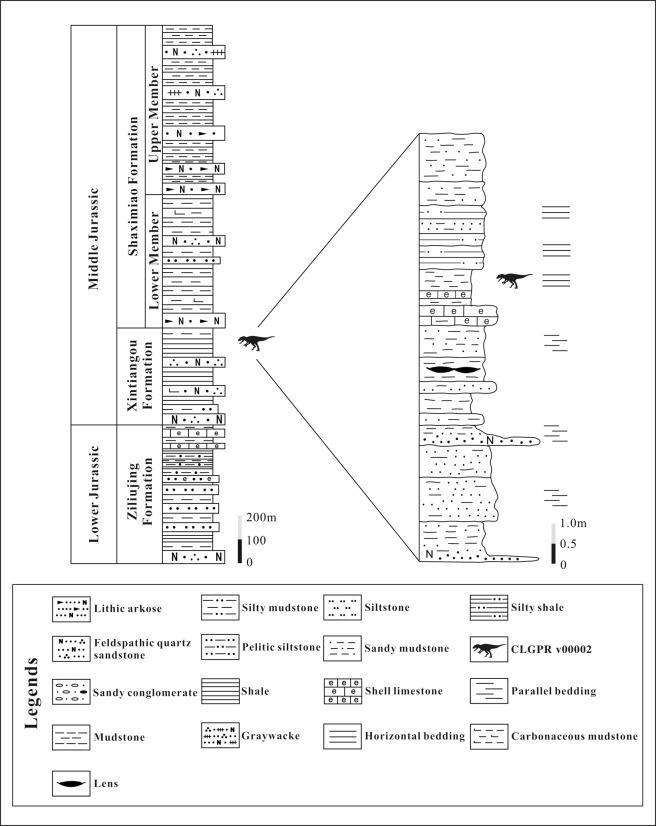
Stratigraphic section of the sedimentary sequence at the type locality. Modified from Li.

The Middle and Upper Jurassic Shaximiao Formation of southwestern China has produced spectacular fossil remains of dinosaurs and many other vertebrates^1,2^. Significant discoveries from this formation in the Chongqing area include the sauropods *Mamenchisaurus hochuanensis*, *Omeisaurus changshouensis*, and the theropod *Yangchuanosaurus shangyouensis*^3^ as well as the stegosaur *Chungkingosaurus jiangbeiensis*^4^. Compared to the fossiliferous Shaximiao Formation, the underlying lower Middle Jurassic Xintiangou Formation^5,6^ has produced few vertebrate fossils. In 2016, we organized a survey in the upper portion of the Xintiangou Formation of Laojun Village, Puan Township, Yunyang City, Chongqing (Fig. 1). This resulted in the discoveries of numerous vertebrate fossils, including a fragmentary theropod specimen recovered from a layer of gray lamellar shell siltstone intercalated with occasional silty mudstone (Fig. 2). The specimen comprises only some presacral vertebrae and several fragmentary bones, but it displays some informative features for its systematic position and provides new information on the early evolution of tetanuran dinosaurs. In the present paper, we establish a new tetanuran species based on this specimen, describe the specimen, and present a short discussion on its implications for the evolution of anatomical character states among early tetanurans.

## Results

Systematic palaeontology

Theropoda Marsh 1881

Tetanurae Gauthier 1986

*Yunyangosaurus puanensis* gen. et sp. nov.

### Etymology

Generic name is a combination of Yunyang (the county-level city in which the type locality is located) and sauros (Greek, reptile). The specific name is derived from Puan (the town name of the type locality).

### Holotype

CLGPR v00002 (Chongqing Laboratory of Geoheritage Protection and Research), a partial skeleton comprising eleven presacral vertebrae, several cervical and dorsal ribs and chevrons. These bones are disarticulated, but were found associated in a small area about 5 square metres, with no other theropod skeletal elements preserved nearby.

### Locality and horizon

Laojun Village, Puan Township, Yunyang City, Chongqing, China (Fig. 2). Middle Jurassic Xintiangou Formation^5,6^. It should be noted that a recent U-Pb dating of the lowest beds of the overlying Shaximiao Formation suggested that the Shaximiao Formation started deposition in late Oxfordian^7^, but that study has not been widely accepted^5^. Furthermore, our newly collected unpublished U-Pb dating data support the traditional view that lower beds of Shaximiao Formation is Middle Jurassic in age.

### Diagnosis

An early-branching tetanuran distinguishable from other tetanurans by the following unique combination of features (*indicates autopomorphies): axial intercentrum sub-triangular in lateral view (sub-rectangular in *Allosaurus* and *Sinraptor*); mediolateral width of axial centrum tapers ventrally, resulting in a subtriangular outline in posterior view*; axis with an accessory medial spinopostzygapophyseal lamina* and a ball-like structure at the base of anterior margin of the axial neural spine*; axis bears very prominent spinopostzygopophyseal lamina similar to non-tetanuran theropods and metriacanthosaurids such as *Sinraptor*, but different from most tetanurans, including other Chinese Jurassic tetanurans and piatnitzkysaurids; cervical centra with flat anterior articular surfaces, similar to non-tetanurans, *Condorraptor*, *Piatnitzkysaurus* and some Jurassic Chinese tetanurans such as ‘*Szechuanosaurus*’ *zigongensis* and *Xuanhanosaurus*, but different from most other non-coelurosaurian tetanurans, which have convex anterior surfaces, including *Leshansaurus*, *Marshosaurus Monolophosaurus* and metriacanthosaurids such as *Sinraptor*; ventral ridge present in anterior cervicals (absent in most tetanurans, but present in some non-tetanurans and the piatnitzkysaurid *Marshosaurus* as a low, anterior eminence); posterior pneumatic foramen or fossa present in some cervical centra (also present in some of the pneumatic centra of *Piatnitzkysaurus* and *Condorraptor* and in all pneumatic centra of most non-tetanuran theropods; but absent in most tetanurans, including Chinese Jurassic tetanurans *Gasosaurus*, *Leshansaurus*, *Sinraptor*, ‘*Szechuanosaurus*’ *zigongensis*, *Xuanhanosaurus* and *Yangchuanosaurus*); prominent epipophyses present in posterior cervicals (absent or relatively low in other Chinese Jurassic tetanurans where known, such as *Monolophosaurus*, *Sinraptor* and ‘*Szechuanosaurus*’ *zigongensis*, as well as *Carnotaurus*, *Torvosaurus*, *Condorraptor* and *Piatnitzkysaurus*); cervical epipophyses prominent and posteriorly oriented* (posterolaterally oriented in most other theropods); posterior cervical and anterior dorsal vertebrae with transversely bifurcated neural spines*, especially prominent in anterior dorsals (less prominent bifurcated neural spines are present in anterior dorsals only in *Condoraptor*, *Marshosaurus* and *Piatnitzkysaurus*; but absent in Chinese Jurassic theropods where preserved, such as *Sinraptor*, *Monolophosaurus*, ‘*Szechuanosaurus’ zigongensis* and *Yangchuanosaurus*); prominent hypapophyses in anterior dorsal vertebrae (also seen in metriacanthosaurids, but small in *Monolophosaurus* and most other early-branching tetanurans); distinctive fossae immediately ventral to dorsal parapophyses*.

#### Description and comparisons

The *Yunyangosaurus puanensis* holotype is inferred to be a sub-adult individual based on the closure of neurocentral sutures in most preserved vertebrae. It has an estimated total body length of 4.7 meters based on the similarity of the measurements of several vertebrae (Table 1) to those of *Sinraptor dongi*^8^. It is therefore similar to species such as *Gasosaurus constructus*^4^ and *Piatnitzkysaurus floresi* from the late Early Jurassic of Argentina^9^. However, it is smaller in body size than most other known early-branching tetanurans from the Jurassic of China^1,8^ with the exception of *Chuandongocoelurus*, the type specimen of which is considerably smaller^10,11^.

**Table 1:** Vertebral measurements (in millimetres) of *Yunyangosaurus puanensis* holotype.

Vert. No.	CL	PH	PW	MW	TH	NSH	NSL	NSW
PV1	58	39	47	14	113	56	34	16
PV 2	69	48	50	15	102+	42+	32	8
PV 3	68	57	64	27	—	—	—	—
PV4	54	54	55+	24	—	—	—	—
PV5	70	59	68	31	106	53	17	10
PV6	58	62	70	29	178	98	20	18
PV7	61	61	68	30	168	85	20	14
PV8	61	54	52+	36	—	—	—	—
PV9	52	61	54+	25	164	90	18	17
PV10	60	60	65	32	—	78+	30	18
PV11	63	57	67	28	151+	71+	37	14

The vertebrae are named as PV1 to PV11 for the convenience of the description; Abbreviations: CL, centrum length; PH, centrum posterior end height; PW, centrum posterior end width; MW, centrum middle portion width; TH, vertebra total height (measured at posterior end); NSH, neural spine height; NSL, neural spine anteroposterior length; NSW, neural spine width; +, incomplete measurements.

Throughout our description, we aim to make comparisons with Chinese Jurassic tetanurans where possible. Among these taxa, *Sinraptor*, *Yangchuanosaurus* and *Shidaisaurus* are metriacanthosaurids^10^ with well-known and relatively similar anatomy to each other^3,12–15^. In general, we have compared *Yunyangosaurus* to *Sinraptor* as an almost complete representative of this clade. Other Chinese Jurassic tetanurans potentially occupy a wide range of phylogenetic positions among early-diverging tetanurans. However, extensive comparisons with some of these are more difficult. The referred materials of some taxa, such as *Chuandongocoelurus* and *Kaijiangosaurus*, are likely represented by specimens from multiple species^10^, so the status of materials that overlap with the preserved material of *Yunyangosaurus* is not clear and requires revision. These taxa are not compared to *Yunyangosaurus* in our description. Nevertheless, they show clear differences: referred vertebral centra of *Chuandongocoelurus* are proportionally elongate, and referred cervical vertebrae of *Kaijiangosaurus* have one pleurocoel on each central side and anteroposteriorly broad and transversely non-bifurcated neural spines^11^. Other taxa such as *Gasosaurus, Leshansaurus*, *‘Szechuanosaurus’ zigongensis* and *Xuanhanosaurus* include overlapping, definitely referable material^16^. Of these, vertebrate of *Leshansaurus*, *‘Szechuanosaurus’ zigongensis* are relatively complete and well-preserved^17^ whereas *Xuanhanosaurus* includes relatively few, incomplete vertebrate^18^ and *Gasosaurus* includes most presacral vertebrae, but they are incomplete and highly reconstructed by plaster, making their original morphology difficult to observe (IVPP)^4^. All four are apparently different from the new taxon, as indicated in our diagnosis and the description below.

Eleven presacral vertebrae of *Yunyangosaurus* are preserved, seven of them are identified as cervical vertebrae, and four as dorsal vertebrae, based on their anatomy (e.g., the position of parapophysis, the orientation of diapophysis, and the presence of a hypapophysis). The cervical series is represented by two anterior cervicals, three middle cervicals, and two posterior cervicals (probably the posteriormost two cervicals). The dorsal series is represented by three anterior dorsal vertebrae (probably the anteriormost ones) and an anterior middle dorsal vertebra.

**Figure 3:**
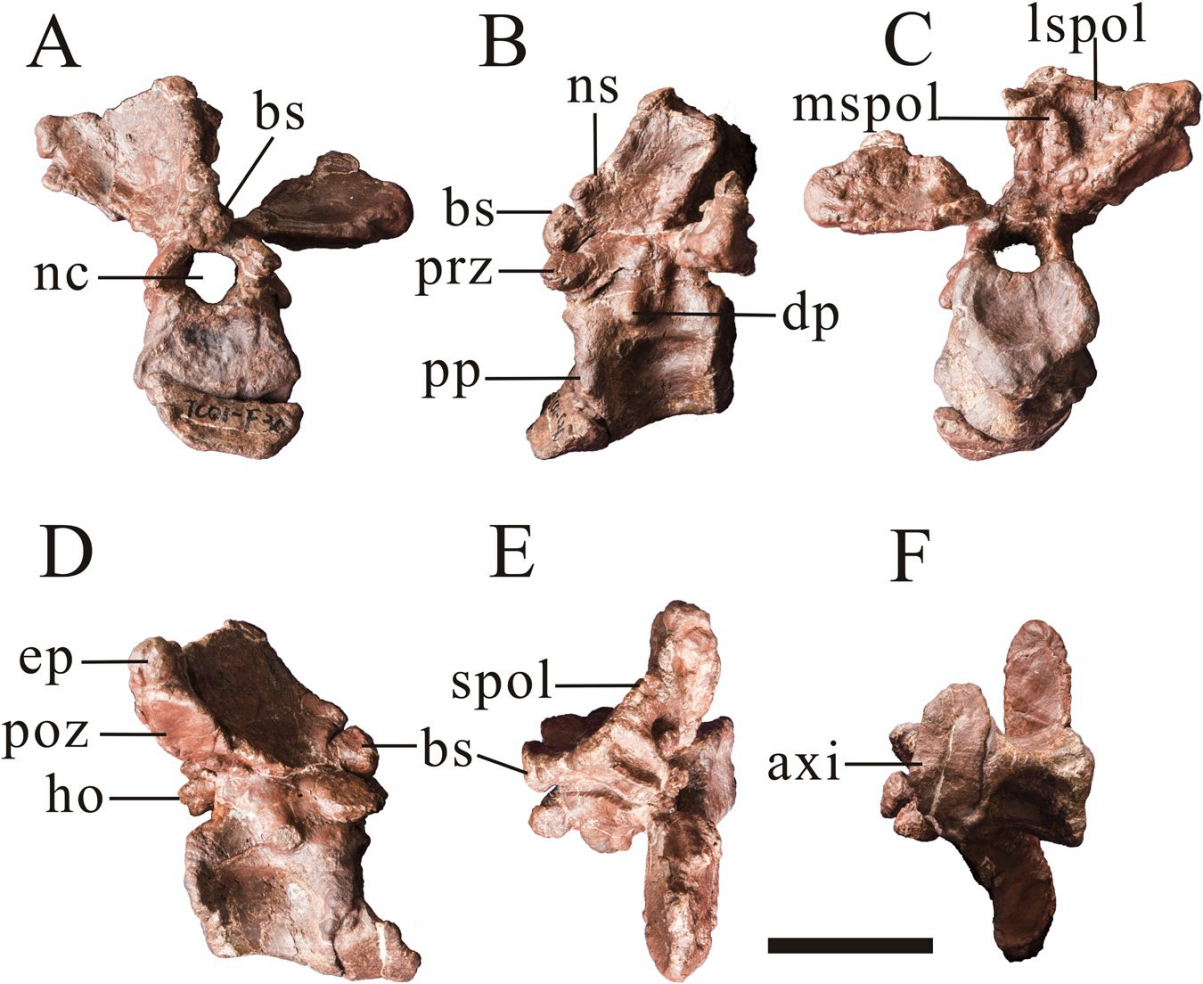
Axis (PV1, Table 1) of *Yunyangosaurus puanensis* holotype in anterior (**A**), left lateral (**B**), posterior (**C**), right lateral (**D**), dorsal (**E**) and ventral. (**F**) Axi, axial intercentrum; bs, ball-like structure; dp, diapophysis; ep, epipophysis; ho, hyposphene; nc, neural canal; ns, neural spine; poz, postzygapophysis; pp, parapophysis; prz, prezygapophysis; lspol, lateral spinopostzygapophyseal lamina; mspol, medial spinopostzygapophyseal lamina. Scale bar = 5 cm.

The axis is mostly preserved, missing the odontoid and part of left spinopostzygapophyseal lamina. The axial intercentrum is attached to the axial centrum, but the line of fusion is obviously visible (Fig. 3A,B). The axial intercentrum is sub-triangular in outline in lateral view. This is similar to the condition in many theropods, including *Leshansaurus*^16^, *Marshosaurus* (CMNH 21704), *Monolophosaurus*^19^, *Piatnitzkysaurus*^9^, ‘*Szechuanosaurus*’ *zigongensis*^17^ and *Neovenator* (Brusatte *et al*., 2008), but is unlike the sub-rectangular axial intercentra of some allosauroids: *Sinraptor*^13^ and *Allosaurus*^20^. The ventral surface of the axial intercentrum is horizontal, as in some non-tetanuran theropods, *Piatnitzkysaurus*^9^ and the possible megalosaurid *Leshansaurus*^16^. In contrast, the ventral surface of the intercentrum is inclined anterodorsally relative to the ventral surface of the axis, such that the two form an oblique angle as in *Monolophosaurus*^19^ and some allosauroids such as *Acrocanthosaurus*^21^, *Giganotosaurus* (IVPP V 7265) and *Sinraptor*^13^.

The axial centrum is platycoelous, with a flat anterior surface. The anterior articular surface of the axial centrum faces anterodorsally, as does the articular surface of the axial intercentrum for the atlantal intercentrum. The axial centrum bears a shallow fossa on its lateral surface on each side. These fossae are asymmetrically developed: the right one is large and shallow, but the left one is small and deep (Fig. 3B,D). This fossa, on the left side, is proportionally larger than those of many other tetanurans, including *Allosaurus*^20^, *Sinraptor*^13^, ‘*Szechuanosaurus*’ *zigongensis*^17^, *Eustreptospondylus*^22^ and *Piatnitzkysaurus* (PVL 4073). The ventral surface of the centrum is transversely narrow with a mediolateral width that tapers ventrally, but nevertheless it is transversely rounded and lacks a distinct ventral keel. This morphology may be an autapomorphy of *Yunyangosaurus* and is unlike the transversely broad ventral surface of the axial centrum in ‘*Szechuanosaurus’ zigongensis* (ZDM 9011), *Yangchuanosaurus hepingensis* (ZDM 0024), *Allosaurus*^20^, *Sinraptor*^13^ and *Eustreptospondylus*^22^. *Piatnitzkysaurus* (PVL 4073) and *Marshosaurus* (CMNH 21704) have transversely narrow axial centra and are therefore somewhat similar to *Yunyangosaurus*. However, the width of the axial centrum does not taper ventrally in these taxa, unlike in *Yunyangosaurus*. The posterior articular surface of the axial centrum of *Yunyangosaurus* has a sub-triangular outline when seeing in posterior view, and in lateral view it forms an acute angle with the ventral margin (Fig. 3C) to a greater degree than in *Yangchuanosaurus*^18^, *Carnotaurus*^23^, *Ceratosaurus*^24^, or *Sinraptor*^13^. The neural canal of the axis is 17 mm high and 18 mm wide while the posterior articular surface is 39 mm high and 47 mm wide, indicating the presence of a relatively large neural canal.

The ventrolaterally oriented axial diapophysis is small and is situated at the fusion line of the centrum and neural arch. The axial prezygapophysis is relatively small and its articular surface faces anterodorsolaterally. The axial postzygapophyses are large with well-developed quadrangular articular surfaces, similar to those of more posterior presacral vertebrae. The articular surfaces face posteroventrally with a lateral inclination of about 20° from horizontal (Fig. 3C). Prominent, broad epipophyses overhang the postzygapophyseal articular surfaces and their long axes are oriented slightly more medially than those of the postzygapophyses. Axial epipophyses are prominent in many non-tetanuran theropods, including *Dilophosaurus*^25^ and *Carnotaurus*^23^, as well as in piatnitzkysaurids (*Piatnitzkysaurus*^9^; *Marshosaurus*, CMNH 21704) and many allosauroids, including metriacanthosaurids such as *Sinraptor*^13^ and *Shidaisaurus*^15^, but are somewhat reduced in megalosaurids (*Eustreptospondylus*)^10,22^ and spinosaurids (*Baryonyx*)^26^.

There are two pairs of spinopostzygapophyseal laminae: a lateral one and a medial one. The prominent lateral one is the major one, and it is comparable to the spinopostzygapophyseal lamina reported in metriacanthosaurids such as *Sinraptor*^13^ and *Shidaisaurus*^15^, and in non-tetanuran theropods such as *Ceratosaurus*^24^ and *Dilophosaurus*^25^. In contrast, it is either small or absent in most early-branching tetanurans (e.g. *Allosaurus*^20^; *Bayronyx*^26^; *Eustreptospondylus*^22^, *Marshosaurus*, CMNH 21704, *Monolophosaurus*^19^ and *Piatnitzkysaurus*^9^). The second, more medially located spinopostzygapophyseal lamina, is oriented vertically and is located within the fossa on the posterior surface of the axial neural spine, between the more lateral spinopostzygapophyseal lamina and the central portion of the neural spine. This medial spinopostzygapophyseal lamina has not been reported in other theropods and may be an autapomorphy of *Yunyangosaurus*.

The neural spine of the axis is posterodorsally oriented (Fig. 3F) as in many other theropods, including *Yangchuanosauru*s *hepingensis* (ZDM 0024), *Allosaurus*^20^, and *Sinraptor*^13^. The anterior margin of the neural spine does not extend anteriorly beyond the prezygapophysis, unlike in other non-tetanuran theropods such as *Coelophysis*^27^, *Dilophosaurus*^25^, *Carnotaurus*^23^, and *Ceratosaurus*^24^. The anterior margin of the neural spine is thickened toward its anteroventral end, and develops into a ball-like structure. This feature has not been reported in other theropods and may be an autapomorphy of *Yunyangosaurus*.

**Figure 4:**
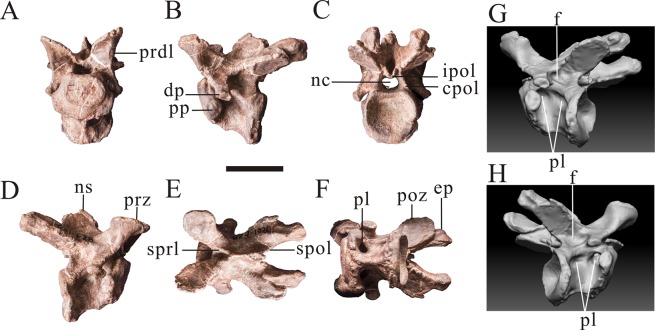
An anterior cervical vertebra (PV2, Table 1) of *Yunyangosaurus puanensis* holotype in anterior (**A**), left lateral (**B**), posterior (**C**), right lateral (**D**), dorsal (**E**), ventral (**F**) and 3D images (**G**,**H**). cpol centropostzygapophyseal lamina; dp, diapophysis; ep, epipophysis; f, fossa; ipol, intrapostzygapophyseal lamina; nc, neural canal; ns, neural spine; pl, pneumatic foramen (pleurocoel); poz, postzygapophysis; pp, parapophysis; prdl, prezygodipophyseal lamina; prz, prezygapophysis; spol, spinopostzygapophyseal lamina; sprl, spinoprezygapophyseal lamina. Scale bar = 5 cm.

The preserved anterior postaxial cervical vertebra is playtcoelous, with an essentially a flat or weakly convex anterior surface. This also occurs in most non-tetanuran theropods^28^, the piatnitzkysaurids *Condorraptor* and *Piatnitzkysaurus*^9,29^ and the Chinese Jurassic tetanurans *‘Szechuanosaurus*’ *zigongensis*^17^, *Xuanhanosaurus*^18^ and figured cervical vertebra of *Chuandongocoelurus* and *Kaijiangosaurus*^11^. In contrast, most other tetanurans have convex anterior surfaces of their cervical centra^28^, including *Monolophosaurus*^19^, megalosaurids such as *Leshansaurus*^16^ and metriacanthosaurids such as *Sinraptor*^13^. The posterior articular surface is concave. The perimeter of the anterior articular surface of the centrum forms a distinct flattened rim (Fig. 4). This condition is widespread among megalosauroids and is a proposed synapomorphy of the group megalosaurids, piatnitzkysaurids and spinosaurids^10,22,26,30^. The centrum is considerably longer anteroposteriorly than dorsoventrally in lateral view, with a length:height ratio of 1.44. It is slightly wider transversely than dorsoventrally in posterior view (Table 1). The centrum is invaded by two pneumatic foramina on each lateral side, an anterior and a posterior foramen. The anterior foramen is deep, penetrating into the body of the centrum. However, the posterior one is shallow in the anterior cervical vertebra, taking the form of a fossa that does not penetrate the centrum. Nevertheless, a deep, penetrating posterior pneumatic foramen is present in some of the other cervical centra (described below). The posterior pneumatic foramen is widely-distributed among non-tetanuran theropods, but is absent in most tetanurans^28,31^. The posterior pneumatic foramen is variably present in some individual cervical and pectoral vertebrae of *Piatnitzkysaurus* (PVL 4073). A well-defined sharp-edged fossa of corresponding size and position to the posterior pneumatic foramen in present in a middle cervical vertebrae of *Condorraptor*^29^. The pneumatic foramina of the anterior cervical centrum of *Yunyangosaurus* are situated at the upper part of the centrum in lateral view, while that are situated at the middle part of the subsequent centra. The ventral surface of the centrum is mediolaterally thin in ventral view and concave in side view, and bear a low, anteroposteriorly oriented ridge.

The neural arch is invaded ventrolaterally by pneumatic fossae as in many theropods, including *Ceratosaurus*^24^, *Torvosaurus*^30,32^, *Piatnitzkysaurus*^9^, *Allosaurus*^24^, *Carnotaurus*^23^, and *Sinraptor*^13^. In *Allosaurus*^20^ and *Monolophosaurus*^19^, the lateral fossae of anterior cervical neural arches are shallow compared to those of *Sinraptor*^13^ and *Yunyangosaurus puanensis*. A horizontal interprezygapophyseal lamina connects the prezygapophyses on the anterior surface of the neural arch. This lamina forms the dorsal roof of the neural canal anteriorly, and the ventral floor of a triangular fossa on the anteroventral surface of the neural arch, together with the spinoprezygapophyseal laminae. The spinopostzygapophyseal laminae and the interpostzygapophyseal laminae bound a much broader, rhomb-shaped fossa on the posterior surface of the neural spine.

The diapophyses are located at the fusion line of the centrum and neural arch. They are inclined ventrolaterally as in other theropods such as *Sinraptor* and; *Piatnitzkysaurus*^9,13^. The articular surface of prezygapophysis is approximately circular, while the articular surface of postzygapophysis is a long oval shape. The epipophyses are prominent, extending well beyond the posterior margins of the postzygapophyses, and they are oriented posteriorly rather than posterolaterally as in most other theropods^9,19,20,30,33^. The presence of prominent and strongly posteriorly-directed epipophyses of the anterior cervical vertebrae may therefore be an autapomorphy of *Yunyangosaurus*. The neural spine is plate-like and posterodorsally oriented, proportionally shorter than in *Ceratosaurus*^24^ and *Allosaurus*^20^. The prespinal and postspinal ridges are prominent.

**Figure 5:**
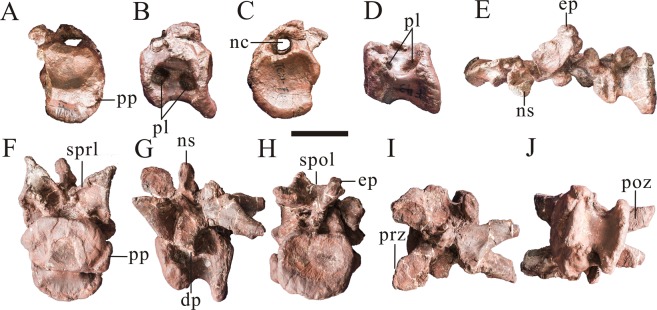
Three middle cervical vertebrae of *Yunyangosaurus puanensis* holotype. The anterior one (PV3, Table 1) in anterior (**A**), left lateral (**B**), in posterior (**C**) and right lateral (**D**) views; the middle one (PV4, Table 1) in dorsal view (**E**); the posterior one (PV5, Table 1) in anterior (**F**), left lateral (**G**), posterior (**H**), right lateral (**I**) and dorsal (**J**) views. cpol centropostzygapophyseal lamina; dp, diapophysis; ep, epipophysis; ipol, intrapostzygapophyseal lamina; nc, neural canal; ns, neural spine; pl, pneumatic foramen (pleurocoel); poz, postzygapophysis; pp, parapophysis; prdl, prezygodipophyseal lamina; prz, prezygapophysis; spol, spinopostzygapophyseal lamina; sprl, spinoprezygapophyseal lamina. Scale bar = 5 cm.

The middle cervical vertebrae are platycoelous (Fig. 5). The centra are slightly longer anteroposteriorly than dorsoventrally in lateral view (Table 1). The posterior articular surfaces are wider transversely than high dorsoventrally, whereas the anterior articular surfaces are smaller, and are higher dorsoventrally than the posterior articular surfaces (Fig. 5B,F) (Table 1). As with the anterior cervicals, the centra are invaded by two pneumatic foramina on each side. But unlike the anterior cervicals, the ventral surfaces of middle cervical vertebrae are broad and flat. The neural arch of the third cervical vertebra is fairly complete, and the upper margin of the neural canal connects the prezygapophyses forming an elongated fossa with the spinoprezygapophyseal laminae in anterior view. The prezygapophysis of the cervical cm3 are large and its articular surfaces face anterodorsolaterally. The postzygapophyses of the cervical cm3 are large with well-developed quadrangular articular surfaces, and prominent epipophyses overhang the postzygapophyses. The neural spines are club-shaped and vertical. They are relatively slender mediolaterally and anteroposteriorly, resulting in a finger-like appearance.

**Figure 6:**
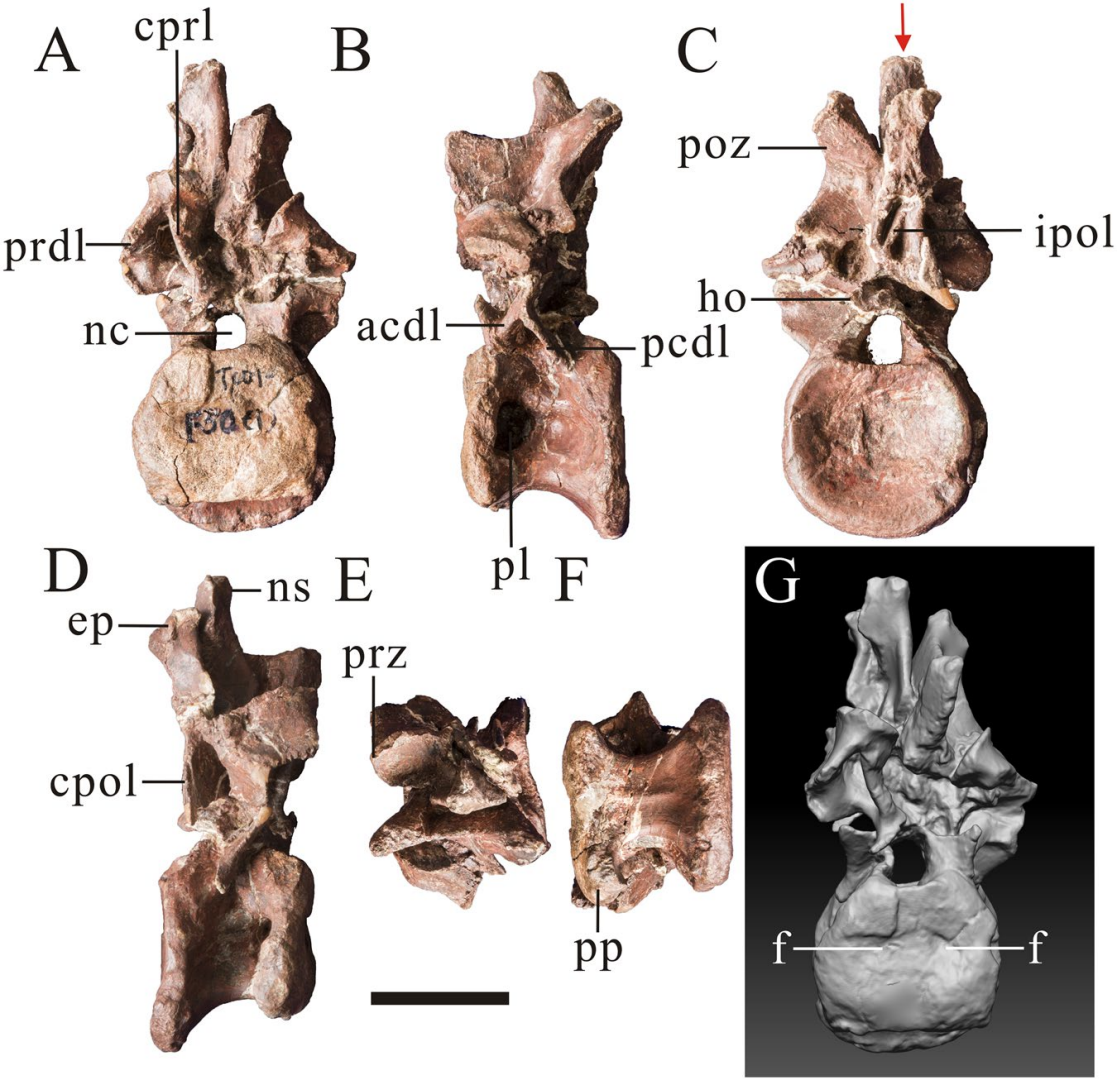
Penultimate cervical vertebra (PV6, Table 1) of *Yunyangosaurus puanensis* holotype in anterior (**A**), left lateral (**B**), posterior (**C**), right lateral (**D**), dorsal (**E**), ventral (**F**) and 3D image (**G**) views. acdl, anterior centrodiapophseal lamina; cpol centropostzygapophyseal lamina; cprl, centroprezygapophyseal lamina; ep, epipophysis; f, fossa; ho, hyposphene; ipol, intrapostzygapophyseal lamina; nc, neural canal; ns, neural spine; pcdl, posterior centrodiapophyseal lamina; pl, pneumatic foramen (pleurocoel); poz, postzygapophysis; pp, parapophysis; prdl, prezygodipophyseal lamina; prz, prezygapophysis. The red arrow directs bifurcation of the neural spine. The red arrow directs bifurcation of the neural spine. Scale bar = 5 cm.

**Figure 7:**
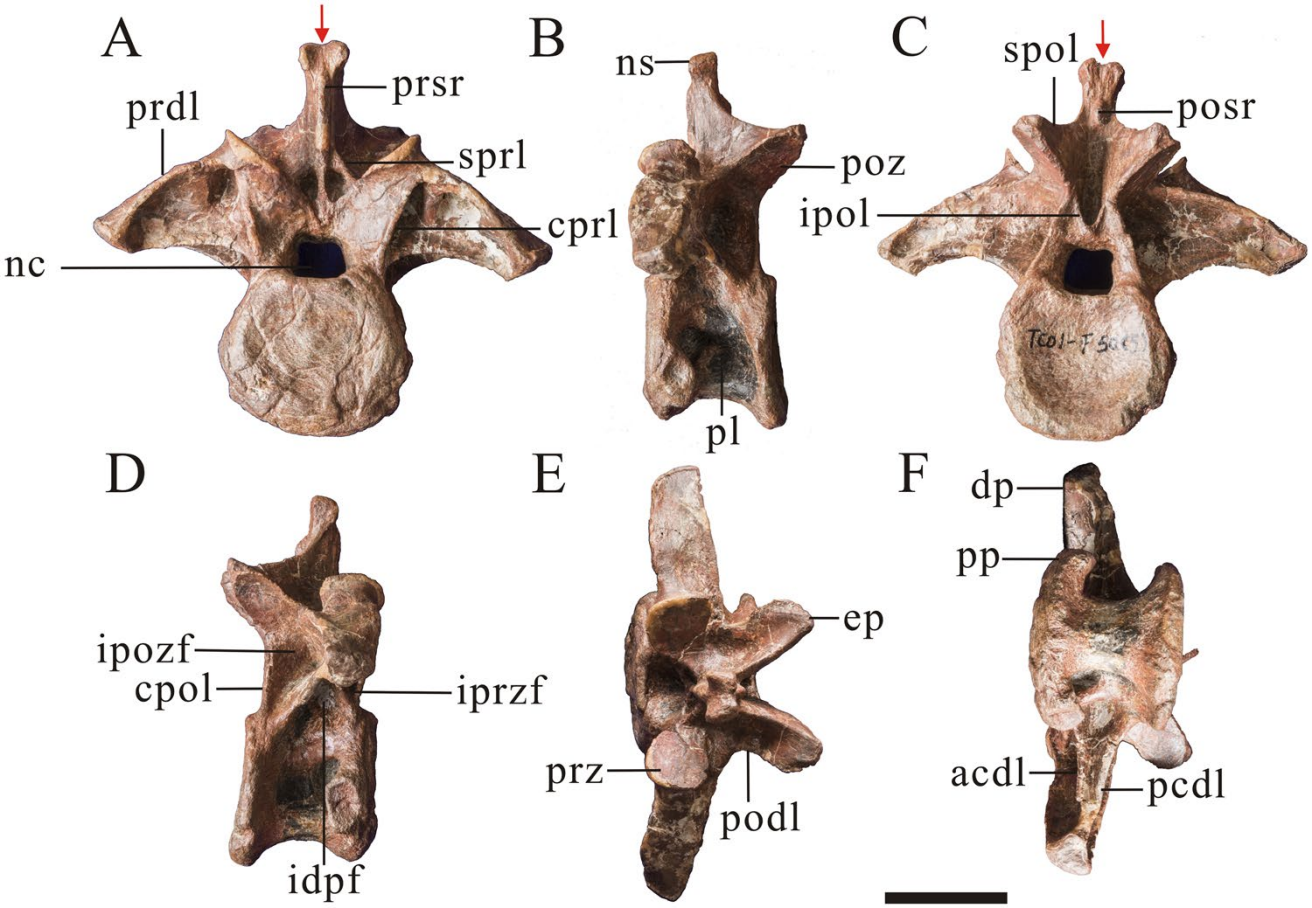
Last cervical vertebra (PV7, Table 1) of *Yunyangosaurus puanensis* holotype in anterior (**A**), left lateral (**B**), posterior (**C**), right lateral (**D**), dorsal (**E**) and ventral (**F**). acdl, anterior centrodiapophseal lamina; cpol centropostzygapophyseal lamina; cprl, centroprezygapophyseal lamina; dp, diapophysis; ep, epipophysis; idpf, infradiapophyseal fossa; ipol, intrapostzygapophyseal lamina; ipozf, infrapostzygapophyseal fossa; iprzf, infraprezygapophyseal fossa; nc, neural canal; ns, neural spine; pcdl, posterior centrodiapophyseal lamina; pl, pneumatic foramen (pleurocoel); podl, postzygadiapophyseal lamina; posr, postspinal ridge; poz, postzygapophysis; pp, parapophysis; prdl, prezygodipophyseal lamina; prsr, prespinal lamina; prz, prezygapophysis; spol, spinopostzygapophyseal lamina; sprl, spinoprezygapophyseal lamina. The red arrows direct bifurcation of the neural spine. Scale bar = 5 cm.

The posterior cervicals are also platycoelous (Figs. 6 and 7). Paired fossae are located ventrally on the anterior surface of the neural spine. These fossae are bounded by the spinoprezygapophyseal laminae laterally and the prezygapophyses ventrally, and are separated on the midline by the prespinal ridge of the neural spine (Fig. 7A). Paired fossae are also present on the posterior surface of the neural arch, bounded ventrally by the dorsal margin of the neural canal, the centropostzygapophyseal laminae and the intrapostzygapophyseal laminae. These fossae are deeper in the penultimate cervical than in the posteriormost one (Figs. 6C and 7C). The centra of the posterior cervicals are invaded by only one pneumatic foramen (the anterior pneumatic foramen) on each lateral side. The posterior pneumatic foramen is absent, unlike in more anterior cervicals. The pneumatic foramina are entirely circular and are deep, penetrating well into the body of the centrum and invading the posterior surfaces of the parapophyses. The articular surfaces of prezygapophyses are approximately circular and dorsolateral, while the articular surfaces of postzygapophyses are long oval and ventrolateral. The epipophyses are extend posteriorly to a level that is slightly posterior to the postzygapophyses. The prespinal and postspinal ridge are more prominent in the posteriormost cervical. The diapophysis originates just dorsal to the level of the neural canal and curves ventrolaterally along its length (Fig. 7F). Three large fossae are located on the lateral surface of the neural arch, ventral to the base of the diapophysis: the infraprezygapophyseal fossa, infradiapophyseal fossa and infrapostzygapophyseal fossa. These fossae are bounded by the prezygodipophyseal lamina, the paraprezygapophyseal lamina, the paradiapophyseal lamina, the posterior centrodiapophyseal lamina, the intrapostzygapophyseal lamina and the postzygadiapophyseal lamina. The infraprezygapophyseal fossa and infrapostzygapophyseal fossa are irregularly quadrilateral, but the infradiapophyseal fossa is triangular (Fig. 7D). In anterior view, the bottom of the right postzygapophyses lamina develop two large and deep fossae divided by a prominent rige, while only one small and deep fossa developed beneath the bottom of the left postzygapophyses lamina. In the posterior view, the bottom of each postzygadiapophyseal lamina develop a deep fossa, and an obvious ridge can be seen beneath the bottom of the right postzygadiapophyseal lamina (Fig. 7A,C). The neural spines of the posterior cervicals are slender in lateral view and their apices are slightly bifurcate (Figs. 6C, 7A,C).

**Figure 8:**
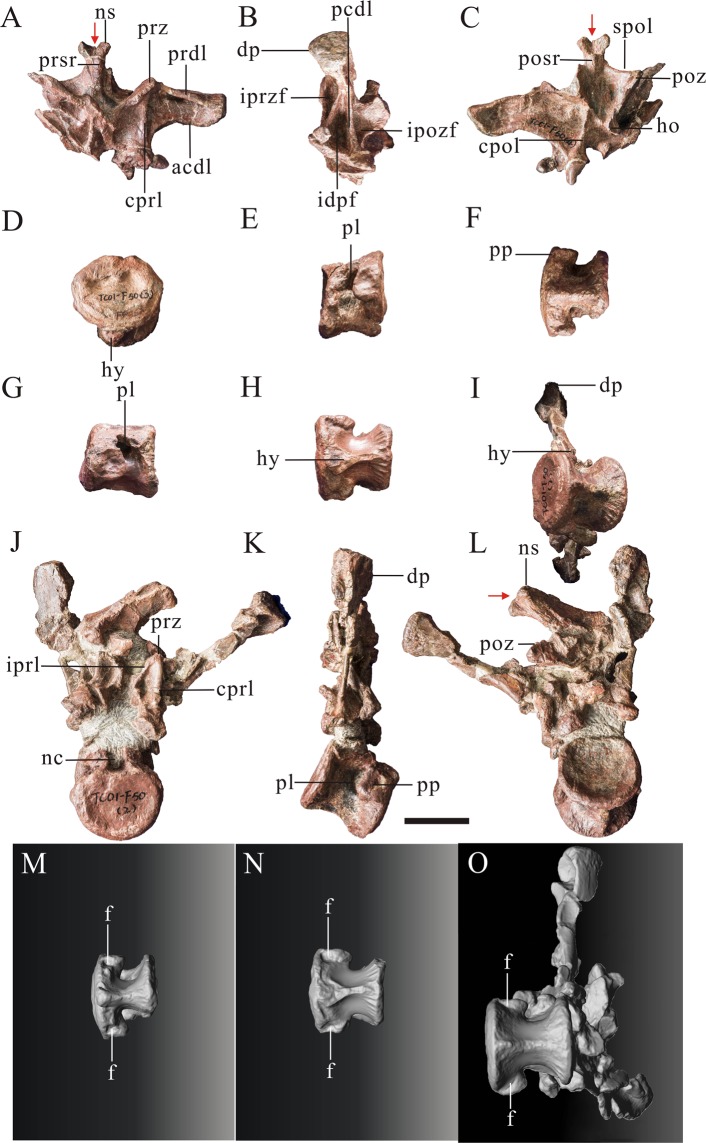
Three anteriormost dorsal vertebrae of *Yunyangosaurus puanensis* holotype. Dorsal 1 (PV9, Table 1) in anterior (**A**), left lateral (**B**), in posterior (**C**); Dorsal 2 (PV8, Table 1) in anterior (**D**), right lateral (**E**),ventral (**F**); Dorsal 3 in left lateral (**G**), ventral (**H**); Dorsal 3 (PV10, Table 1) in ventral (**I**), anterior (**J**), right lateral (**K**), posterior (**L**) and 3D images (**M**–**O**). acdl, anterior centrodiapophseal lamina; cpol centropostzygapophyseal lamina; cprl, centroprezygapophyseal lamina; dp, diapophysis; f, fossa; hy, hypapophysis; ho, hyposphene; idpf, infradiapophyseal fossa; ipozf, infrapostzygapophyseal fossa; iprzf, infraprezygapophyseal fossa; iprl, intraprezygapophyseal lamina; nc, neural canal; ns, neural spine; pcdl, posterior centrodiapophyseal lamina; pl, pneumatic foramen (pleurocoel); posr, postspinal ridge; poz, postzygapophysis; pp, parapophysis; prdl, prezygodipophyseal lamina; prsr, prespinal rige; prz, prezygapophysis; spol, spinopostzygapophyseal lamina. The red arrows direct bifurcation of the neural spine. Scale bar = 5 cm.

**Figure 9:**
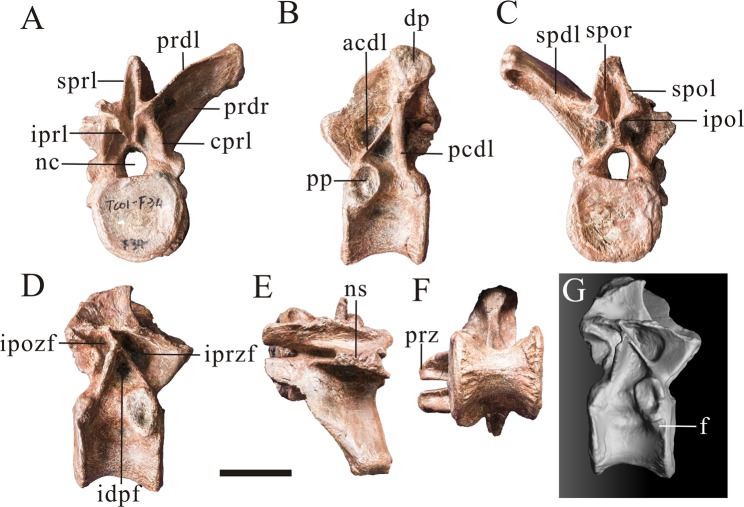
An anterior middle dorsal vertebra (PV11, Table 1) of *Yunyangosaurus puanensis* holotype in anterior (**A**), left lateral (**B**), posterior (**C**), right lateral (**D**), dorsal (**E**), ventral (**F**) and 3D image (**G**). acdl, anterior centrodiapophseal lamina; cprl, centroprezygapophyseal lamina; dp, diapophysis; f, fossa; idpf, infradiapophyseal fossa; ipol, intrapostzygapophyseal lamina; ipozf, infrapostzygapophyseal fossa; iprl, intraprezygapophyseal lamina; iprzf, infraprezygapophyseal fossa; nc, neural canal; ns, neural spine; pcdl, posterior centrodiapophyseal lamina; pl, lateral depression (pleurocoel); pp, parapophysis; prdl, prezygodipophyseal lamina; prdr, prezygodiapophyseal ridge; prz, prezygapophysis; spdl, spinodiapophyseal lamina; spol, spinopostzygapophyseal lamina; spor, spinspinopostzygapophyseal ridge; sprl, spinoprezygapophyseal lamina. Scale bar = 5 cm.

The three anteriormost dorsal vertebrae of *Yunyangosaurus* have platycoelous centra. This is similar to the condition in most theropods, although the first four or five dorsals of *Acrocanthosaurus* may be opisthocoelous^34^. The ratio of the anteroposterior length of centrum to the height of the posterior articular surface of centrum is 0.85 to 1.13 among anterior dorsal vertebrae of *Yunyangosaurus*, and the ratio of height to width of the posterior articular surface of centrum is 0.85 to 0.92. The anterior articular surfaces of the centra are marked by a well-developed circumferential groove, and two shallow fossae are also present on the anterior articular surfaces of all dorsals, located just dorsal to mid-height (Figs. 8D,J and 9A). The anterior articular surfaces of the dorsals are roughly circular and posterior articular surfaces are nearly square.

The central portion of each dorsal centrum is transversely constricted relative to the articular surfaces. Distinct, single pneumatic foramina invade the lateral surfaces of the centra posterior to the parapophyses in anterior dorsals. Pneumatic foramina are present only in the cervical and anterior dorsal centra of *Yunyangosaurus*, and are absent from more posterior dorsals. This is similar to the condition in many Jurassic theropods^35^, for example, the posteriormost centrum to bear a pneumatic foramen is the 14^th^ presacral of *Sinraptor*^13^ and the 13^th^ presacral of *Allosaurus*^20^. Nevertheless, pneumatic foramina in more posterior dorsal centra are present in geologically younger members of many groups of theropods and take various morphologies^35^. For example, large pneumatic openings are found in all dorsal centra of *Torvosaurus*^30^. There is one conspicuous fossa beneath each parapophysis in all of the preserved dorsals of *Yunyangosaurus*. Radial grooves are present on the posteroventral surface of the centrum in anterior dorsals (Fig. 8H,I). The hypapophyses are also prominent in the anterior dorsals. Prominent hypoapophyses are widely-distributed among adult tetanurans, including *Allosaurus*^20^, *Condoraptor*^29^, *Marshosaurus* (CMNH 21704), *Sinraptor*^13^, *Piatnitzkysaurus*^9^ and *Streptospondylus*^36^. The most prominent hypapophysis of *Allosaurus* is in the second dorsal (Madsen, 1976), which is the 11^th^ presacral vertebra, as in *Sinraptor*^13^.

The hyposphenes are large in the anterior dorsals and have a roughly constant transverse width dorsoventrally. This differs from the ventrally-wide, triangular hyposphene morphology that is found among many early-diverging tetanurans^37^. Step-like ridges are present lateral to the hyposphenes, running posterodorsally from the dorsal border of neural canal to the posterior edge of postzygapophyses (Figs. 6C and 8C). These ridges have a homoplastic distribution among early-diverging tetanurans being present both among meaglosauroids and in metriacanthosaurids^37^. The spinopostzygapophyseal lamina extends posterodorsally to the posterolateral margin of the neural spine from the posterior corner of the postzygapophysis. The prespinal and postspinal ridges are developed in the anterior dorsals (Fig. 8A,C). The spinopostzygapophyseal laminae are developed in the anterior dorsals. The articular surface of the diapophysis is trapeziform and slightly concave (Fig. 8B). Below the base of the diapophysis, there are three large fossae called infraprezygapophyseal fossa, infradiapophyseal fossa and infrapostzygapophyseal fossa, which on the lateral surface of the base of the neual arch. These fossae are formed by the prezygodipophyseal lamina, the paraprezygapophyseal lamina, the paradiapophyseal lamina, the posterior centrodiapophyseal lamina, the intrapostzygapophyseal lamina and the postzygadiapophyseal lamina. The infraprezygapophyseal fossa and infrapostzygapophyseal fossa are irregularly quadrilateral, but the infradiapophyseal fossa is triangular (Fig. 8B). The bottom of the prezygodipophyseal laminae develop two small ridges (one is the prezygodiapophyseal ridge) and some small shallow fossae in the anterior dorsal. The neural spine is claviform in the anterior dorsals and the tops of the anterior dorsals are transversely bifurcated as in the posterior cervicals, but the bifurcation is deeper than in the latter. The articular surface of prezygapophyses are approximately circular, while the articular surface of postzygapophyses are oval.

The centrum of the preserved anterior middle dorsal is amphiplatyan. The amphiplatyan condition is present in all dorsals of *Carnotaurus*^23^, whereas it is only present from the fifth dorsal (14^th^ presacral) and more posteriorly in *Allosaurus*^20^ and from the sixth (15^th^ presacral) of *Monolophosauurus*^19^. Clear, radial grooves can be found in the anteroventral and posteroventral portions of the centrum (Fig. 9F). A shallow depression is present on the lateral surface of this centrum in *Yunyangosaurus*. This may be homologous to the large, extensive, fossa-like pneumatic foramina that are present in the anterior dorsal centra of some megalosaurids such as *Torvosaurus* and *Eustreptospondylus*^10,22,30^, though it is relatively shallow, and not pronounced as in those taxa.

The neural canal of the anterior middle dorsal is 11 mm high and 8 mm wide while the anterior articular surface is 23 mm high and 25 mm wide, so the neural canal is about 1/3 to 1/2 as big as the centrum (Fig. 9A). The hypantrum is highly developed and the ridges of the middle portion between the dorsal margin of the neural canal and the junction of the prezygapophyses are developed. These ridges are called intraprezygapophyseal laminae. They contact the articular surfaces of the prezygapophyses, forming two triangular fossae with the margin of the neural canal and anterior centroprezygapophyseal laminae (Fig. 9A). The hyposphene is smaller in the anterior middle dorsal than in the anterior dorsals. A ridge is visible on the spinopostzygapophyseal lamina of the anterior/middle dorsal, while it is missing in the anterior dorsal (Fig. 9C). The spinoprezygapophyseal and spinopostzygapophyseal laminae are developed in the anterior middle dorsal. The spinoprezygapophyseal lamina is obvious in the anterior middle dorsal but only a gentle ridge in the anterior dorsals (Fig. 9A). The diapophyses are upswept and below the base of which are three large fossae called infraprezygapophyseal fossa, infradiapophyseal fossa and infrapostzygapophyseal fossa, on the lateral surface of the base of the neual arch. These fossae are formed by the prezygodipophyseal lamina, the paraprezygapophyseal lamina, the paradiapophyseal lamina, the posterior centrodiapophyseal lamina, the intrapostzygapophyseal lamina and the postzygadiapophyseal lamina. The infraprezygapophyseal fossa and infrapostzygapophyseal fossa are irregularly quadrilateral, but the infradiapophyseal fossa is triangular (Fig. 9D). In the infrapostzygapophyseal fossa, the centropostzygapophyseal ridge is visible in the anterior middle dorsal but illegible in the anterior dorsals (Fig. 9B). The neural spine is incompletely preserved, but it is inferred to be plate-like (Fig. 9A).

Two proximal portions of the cervical ribs are preserved. The capitulum and tuberculum are widely separated, and the articular facets lie in the same plane, so presumably they belong to posterior cervicals. Deep fossae are found between the base of the capitulum, tuberculum, and prominent anterolateral process. The angle between the capitulum and tuberculum is about 70°. Some partial dorsal ribs are preserved. The capitulum and tuberculum are more widely separated, and the proximal pneumatopore is absent. The cross-sectional shapes of the ribs are subcircular.

**Figure 10:**
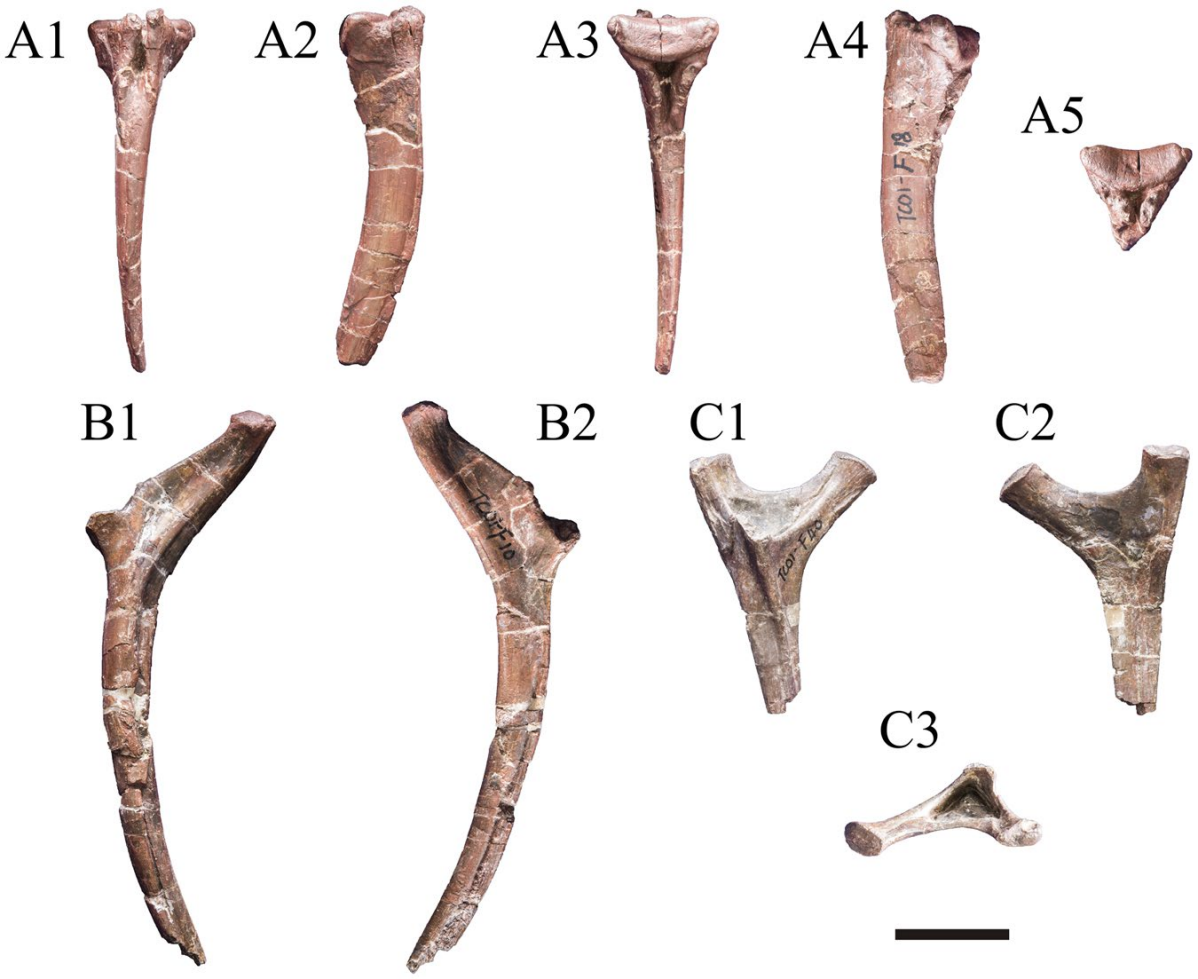
A partial chevron (A1-A5) and two ribs (B1-B2 are dorsal ribs, C1-C3 are cervical ribs) of *Yunyangosaurus puanensis* holotype.

Three unforked chevrons with bridged haemal canal are preserved, and the haemal cannal is short approximately 20% chevron length (Fig. 10).

## Discussion

*Yunyangosaurus puanensis* is represented by limited material, but it displays numerous informative features for its systematic position and taxonomy. It is highly likely a tetanuran dinosaur based on the general morphology of the presacral vertebrae, and in particular several tetanuran or orinoidan synapomorphies are present^10,38^: the axial neural spine is narrow anteroposteriorly and somewhat rod-like; the prezygapophyses of anterior cervical vertebrae are enlarged and situated entirely lateral to the neural canal; and there is a pronounced ventral keel in the anterior dorsal vertebrae.

However, *Yunyangosaurus puanensis* also lacks several derived features seen in most other early-branching tetanurans^10,38^. These features include strongly opisthocoelous cervical centra, cervical vertebrae with only one pair of pneumatic foramina (rather than two, including both the anterior and posterior pneumatic foramen, as in most non-tetanuran theropods), and the presence of prominent axial spinopostzygapophyseal laminae. Although the anterior surfaces of the anterior cervical centra of *Yunyangosaurus puanensis* are weakly convex, this is unlike the prominent, convexity seen in the opisthocoelous condition that is widespread among early-diverging tetanurans. Furthermore, the middle and posterior cervical vertebrae of *Yunyangosaurus* are strictly platycoelous with flat anterior surfaces. Among tetanurans, this morphology, with flat to weakly convex anterior surfaces of the cervical centra, is also present in ‘*Szechuanosaurus’ zigongensis* (ZDM 9011), the piatnitzkysaurid *Piatnitzkysaurus*^9^, *Xuanhanosaurus*^18^ and *Condorraptor*^39^. Furthermore, the presence of a second, posterior, pneumatic formamen is variably present in some cervical centra of *Piatnitzkysaurus*^35^, although it is much more prominently and consistently developed in *Yunyangosaurus*, similar to the condition in most non-tetanuran theropods^28^. The axial spinopostzygapophyseal lamina is reduced in most tetanurans, unlike in *Yunyangosaurus*. Among tetanurans, a prominent axial spinopostzygapophyseal lamina is also present in metriacanthosaurids such as *Sinraptor*^13^.

*Yunyangosaurus puanensis* also lacks an axial intercentrum with an anterodorsally inclined ventral surface. This feature is present in many early-branching tetanurans including *Monolophosaurus*^19^ and *Sinraptor*^13^. However, the ventral surface of the axial intercentrum of *Yunyangosaurus* is horizontal similar to the condition in some megalosauroids, such as *Leshansaurus* and *Piatnitzkysaurus*^9,16^.

**Figure 11:**
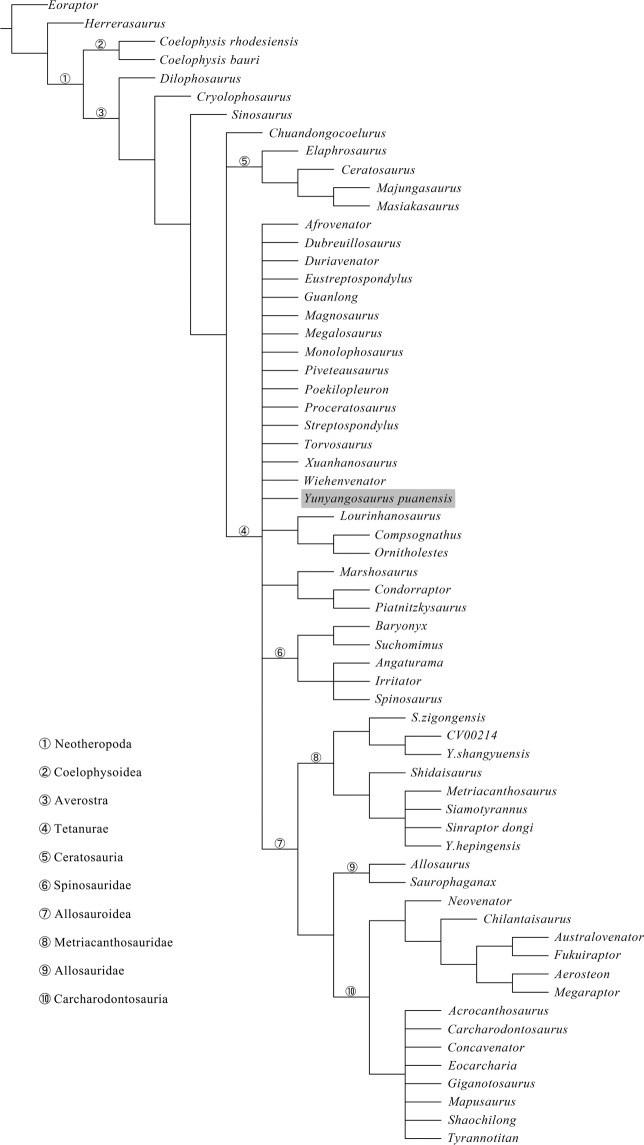
Strict consensus of 4860 most parsimonious trees produced by our analysis showing the systematic position of *Yunyangosaurus puanensis*.

**Figure 12:**
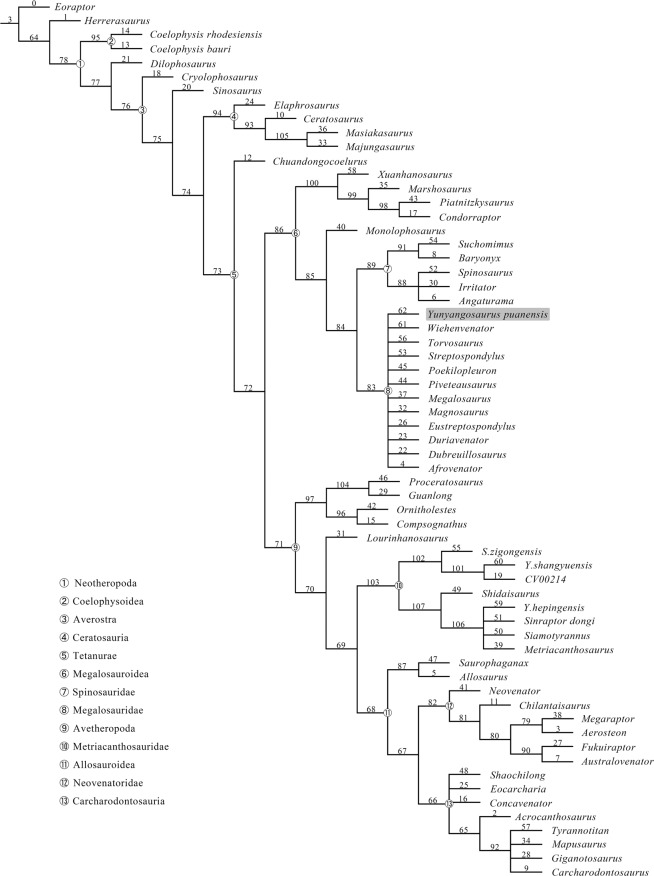
Strict consensus of 1107 most parsimonious trees produced by our parsimonious analysis with implied weighting showing the systematic position of *Yunyangosaurus puanensis*.

*Yunyangosaurus* possesses several features that are only known in some megalosauroids, especially piatnitzkysaurids (described above). These also include the presence of a prominent rim round articular surface of cervicals^10^ and bifurcated anterior dorsal neural spines (present in piatnitzkysaurids)^7^. A numerical cladistic analysis was then ran to assess this phylogenetic hypothesis (see Methods), but which fails to place *Yunyangosaurus* within the Megalosauroidea and instead posits it in an unresolved position with many other tetanurans (Fig. 11). Noteworthy is also that the inclusion of *Yunyangosaurus* in the dataset led to collapsing of the Megalosauroidea and the Coelurosauria. This suggests the complex combination of character states in *Yunyangosaurus* has an impact on the tetanuran phylogeny, which is still far from robust for understanding the evolution of the group. However, we reran the analysis using implied weighting (see Methods), and this analysis places *Yunyangosaurus* within the Megalosauroidea in an unresolved position with many other megalosauroids (Fig. 12).

The discovery of *Yunyangosaurus* has implication for our understanding of character evolution of tetanuran theropods. For example, *Yunyangosaurus* displays several features that are generally absent in most other tetanurans, and these features include the presence of posterior pneumatic foramina throughout the cervical series and in particular pneumatic foramen in middle cervical centra (also present occasionally in some cervical centra of piatnitzkysaurids), prominent spinopostyzgopophyseal laminae (also present in metriacanthosaurids), and flat anterior articular surfaces of the cervical centra (also present in piatnitzkysaurids).

However, currently our knowledge of *Yunyangosaurus* is relatively incomplete, preventing us from attaining a more complete understanding of its affinities among early tetanurans. For now, *Yunyangosaurus* serves to highlight the potentially complex character distribution of character states of the axial column among early-branching tetanurans, and especially among Chinese Jurassic tetanurans.

## Methods

### Phylogenetic analysis

We added *Yunyangosaurus* into a comprehensive dataset for tetanuran phylogeny^40^, and the final matrix includes 351 characters and 63 operational taxonomical units (OTUs). The matrix was analyzed using TNT 1.5^41^, with the ‘New Technology’ search option, using a driven search that stabilized consensus twice with a factor of 25, and default settings for sectorial, ratchet, tree drift and tree fusion. The resulting 142 most parsimonious trees (MPTs) with a tree length = 1103 step, a CI = 0.39, and a RI = 0.67 were then subjected to tree bisection and reconnection (TBR) branch swapping, and finally 4860 MPTs were found. The strict consensus of these 4860 MPTs places *Yunyangosaurus* at the base of the Tetanurae in an unsolved position with several other early-branching tetanuran theropods. However, we reran the analysis using implied weighting parsimony with a k value of 8.0, and this analysis places *Yunyangosaurus* within the Megalosauroidea in an unresolved position with many other megalosauroids (Fig. 12).

## Supplementary information

Supplementary Information
