# Supplementary material for: A new possible megalosauroid theropod from the Middle Jurassic Xintiangou Formation of Chongqing, People’s Republic of China and its implication for early tetanuran evolution
Source: Sci Rep. 2020 Jan 10;10:139. doi: 10.1038/s41598-019-56959-x (PMC6954265; doi:10.1038/s41598-019-56959-x)
Supplement: Supplementary file 1 — Supplementary Information [file 41598_2019_56959_MOESM1_ESM.pdf]

A new possible megalosauroid theropod from the Middle Jurassic Xintiangou Formation of Chongqing, People's Republic of China and its implication for early tetanuran evolution

Hui Dai[1,*], Roger Benson[2], Xufeng Hu[1], Qingyu Ma[1], Chao Tan[1], Ning Li[1], Ming Xiao[1], Haiqian Hu[1], Yuxuan Zhou[1], Zhaoying Wei[1], Feng Zhang[1], Shan Jiang[3], Deliang Li[4], Guangzhao Peng[3], Yilun Yu[5,6], Xing Xu[5,6,*]

[1]Chongqing Laboratory of Geoheritage Protection and Research, No. 208 Hydrogeological and Engineering Geological Team, Chongqing Bureau of Geological and Mineral Resource Exploration and Development, Chongqing 400700, China

[2]Department of Earth Sciences, University of Oxford, Oxford OX1 3AN, UK

[3]Zigong Dinosaur Museum Zigong 643013, Sichuan, China

[4]Chongqing Institute of Geological Survey, Chongqing 401122, China

[5]Key Laboratory of Vertebrate Evolution and Human Origins of Chinese Academy of Sciences, Institute of Vertebrate Paleontology and Paleoanthropology, Chinese Academy of Sciences Beijing 100044, China

[6]CAS Center for Excellence in Life and Paleoenvironment, Beijing, China

*Authors for correspondence

Hui Dai, e-mail: dhui6251@qq.com

Xing Xu, email: xu.xing@ivpp.ac.cn

Appendix

1. Character coding of *Yunyangosaurus puanensis* based on the matrix published by Rauhut et al., (2016).

???

??01101??010000

0000001121?000011011100?0?1????????????00?????0111???????????????????????????????

???

?????????????????

References

Rauhut, O. W., Hübner, T. & Lanser, K.-P. A new megalosaurid theropod dinosaur from the late Middle Jurassic (Callovian) of north-western Germany: Implications for theropod evolution and faunal turnover in the Jurassic. *Palaeontologia Electronica* 19, 1-65 (2016).
